# Cytotoxicity of methanol extracts of *Annona muricata*, *Passiflora edulis* and nine other Cameroonian medicinal plants towards multi-factorial drug-resistant cancer cell lines

**DOI:** 10.1186/s40064-016-3361-4

**Published:** 2016-09-27

**Authors:** Victor Kuete, Joachim K. Dzotam, Igor K. Voukeng, Aimé G. Fankam, Thomas Efferth

**Affiliations:** 1 Department of Pharmaceutical Biology, Institute of Pharmacy and Biochemistry, University of Mainz, 55128 Mainz, Germany; 2 Department of Biochemistry, Faculty of Science, University of Dschang, P.O. Box 67, Dschang, Cameroon

**Keywords:** *Annona muricata*, Apoptosis, Cameroon, Cytotoxicity, Medicinal plants, *Passiflora edulis*

## Abstract

**Background:**

Cancer cells rapidly acquire resistance leading to treatment failures. In the present study, we have evaluated the cytotoxicity of 17 methanol extracts from 11 Cameroonian medicinal plants against the sensitive leukemia CCRF–CEM cells and the best ones were further tested on a panel of 8 other human cancer cell lines, including various MDR phenotypes as well as against the normal AML12 hepatocytes.

**Methods:**

The cytotoxicity of the extracts was determined using a resazurin reduction assay meanwhile flow cytometry was used to measure cell cycle, apoptosis, mitochondrial membrane potential (MMP), and reactive oxygen species.

**Results:**

In an initial screening using leukemia CCRF–CEM cells, ten extracts from five plants namely *Alchornea floribunda*, *Annona muricata*, *Euphorbia prostata*, *Pachypodanthium staudtii* and *Passiflora edulis* displayed IC_50_ values below 20 µg/mL. They were further tested in 8 other cell lines as well as in normal AML12 hepatocytes. All selected extracts were active against leukemia CEM/ADR5000 cells with IC_50_ value below 40 µg/mL. IC_50_ values ranging from 10.13 µg/mL (towards CEM/ADR5000 cells) to 72.01 µg/mL [towards resistant colon carcinoma HCT116 (*p53*^−*/*−^) cells] for *Pachypodanthium staudtii* roots and from 0.11 µg/mL (towards CCRF–CEM cells) to 108 µg/mL (towards P-glycoprotein-over-expressing CEM/ADR5000 cells) for doxorubicin were obtained in the eight other cancer cell lines studied. Extracts from *Annona muricata* leaves (AML) and seeds (AMS), and *Passiflora edulis* fruit (PEF) had IC_50_ values below 1 µg/mL against CCRF–CEM cells and below 10 µg/mL against its MDR subline CEM/ADR5000 cells. AML, AMS and PEF induced MMP-loss-mediated apoptosis in CCRF–CEM cells.

**Conclusions:**

Results of the present study suggest that some of the tested plants namely *Alchornea floribunda*, *Annona muricata*, *Euphorbia prostata*, *Pachypodanthium staudtii* and *Passiflora edulis* represent a source of anticancer drugs. *Annona muricata* and *Passiflora edulis* are good cytotoxic plants that could be exploited to develop phytomedicine to fight mostly hematological cancers including MDR phenotypes.

## Background

The development of resistance to cytotoxic agents represents a major concern in cancer chemotherapy. Multi-drug resistance (MDR) is associated with over-expression of transmembrane glycoprotein (P-gp) which functions as a drug efflux pump, reducing the intracellular levels of cytotoxic drugs (Juranka et al. 1989). P-gp belongs to the ATP-binding cassette (ABC) transport proteins, which also include the multi-drug resistance associated protein 1 (MRP1) (Shen et al. 2011; Biedler and Spengler 1994; Efferth et al. 2003a), or the breast cancer resistance protein (BCRP/ABCG2) (Shen et al. 2011). The oncogene epidermal growth factor receptor (EGFR) (Biedler and Spengler 1994; Efferth et al. 2003a, b) and the deletions or inactivation of tumor suppressor gene p53 (el-Deiry 1997) have also been involved in MDR mechanism of cancer cells. Overcoming this resistance requires a permanent search of new antineoplastic agents. In the past, natural products from plant kingdom have revealed a high potential as cytotoxic drug reservoir (Kuete and Efferth 2011). According to the World Health Organization, about 80 % of the population of developing countries relies on traditional medicines, mostly plant drugs, for their primary health care needs (FAO 1997). It has also been reported that modern pharmacopoeia still contain at least 25 % drugs derived from plants and many others which are synthetic analogues (FAO 1997). Therefore, fighting cancers with botanicals represents a very promising alternative, especially regarding the diversity of plant’s secondary metabolites. African flora has previously been found to be very fruitful in the search of antiproliferative molecules. Many compounds including xanthones: 8-hydroxycudraxanthone G, morusignin I, cudraxanthone I (Kuete et al. 2013a), and xanthone V1 (Kuete et al. 2011a), benzophenones: guttiferone E and isogarcinol (Kuete et al. 2013b), quinone: 2-acetylfuro-1,4-naphthoquinone (Kuete et al. 2011a), flavonoids: 4-hydroxylonchocarpin, 6,8-diprenyleriodictyol (Kuete et al. 2011b), 2′,4′-dihydroxy-3′,6′-dimethoxychalcone and 4′-hydroxy-2′,6′-dimethoxychalcone (Kuete et al. 2014a; Dzoyem et al. 2012) and alkaloids: isotetrandrine (Kuete et al. 2015a) and montrofoline (Kuete et al. 2015b) displayed good antiproliferative effects against various cancer cell lines. In a collaborative research programme between the Council for Scientific and Industrial Research (CSIR) in South Africa and the National Cancer Institute (NCI) in the USA, several South African plant extracts exhibited anticancer activity against a panel of three human cell lines (breast MCF7, renal TK10 and melanoma UACC62) (Fouche et al. 2006, 2008). African medicinal plants such as *Fagara heitzii* (Dzoyem et al. 2013), *Echinops giganteus*, *Xylopia aethiopica*, *Piper capense*, *Imperata cylindrica* (Kuete et al. 2011c), *Beilschmiedia acuta*, *Clausena anisata* (Kuete et al. 2013c) also displayed good cytotoxicity towards drug-sensitive and drug-resistant cancer cell lines. In our ongoing search of anticancer products from African medicinal flora, we designed the present study to investigate the cytotoxicity of 11 plants traditionally used to manage cancer or disease states bearing relevance to cancer or cancer-like symptoms, such as immune and skin disorders, inflammatory, infectious, parasitic and viral diseases (Kuete et al. 2015a). The study was extended to the evaluation of the ability of the three most active extracts from two medicinal plants, *Annona muricata* Lin. (Annonaceae) and *Passiflora edulis* Sims (Passifloraceae) to alter the cell cycle distribution, caspases activity, mitochondrial membrane potential (MMP) and to increase the production of reactive oxygen species (ROS) in leukemia CCRF–CEM cells.

## Methods

### Plant material and extraction

All medicinal plants tested are traditionally used in the management of cancer or disease states with symptoms related to cancer. Plants were collected in different regions of Cameroon in January 2012. They included *Pachypodanthium staudtii*, *Alchornea floribunda*, *Annona muricata*, *Canarium schweinfurthii*, *Hibiscus esculentus*, *Colocasia esculenta*, *Moringa oleifera*, *Triumphetta pentandra*, *Xanthosoma mafaffa*, *Euphorbia prostata* and *Passiflora edulis*. The plant parts investigated are shown in Table 1. The plants were identified at the National Herbarium (Yaoundé, Cameroon), where voucher specimens were deposited under the reference numbers shown in Table 1. The air-dried and powdered plant material (50 g) was soaked in methanol (200 mL) for 48 h, at room temperature. The methanol extract was concentrated in vacuum under reduced pressure at 68 °C to give the crude extract. This extract was completely dried at room temperature, then conserved at 4 °C until further use.

**Table 1:** General information and reports on evidence of biological activities and chemistry of the studied plants

Species (family); voucher number^a^	Traditional uses	Parts used (%yield)^b^	Bioactive or potentially bioactive components	Bioactivity of crude extract
*Alchornea floribunda* Müll. Arg. (Euphorbiaceae) 4595/HNC	Treatment of bacterial and parasitic infections, painful urination in children (Adjanohoun et al. 1996; Jiofack et al. 2009), urinary, respiratory and intestinal problems, pains in the heart, diarrhoea, ovarian problems, stomach ailments and intestinal disorders (Siwe Noundou et al. 2014), trypanosomiasis, urinary, respiratory and intestinal disorders (Musuyu Muganza et al. 2012; Mesia et al. 2008), inflammation (Okoye et al. 2011)	Bark (18.91 %) and leaves (4.56 %)	Eugenol, cadinol, nanocosaine, ethyl iso-allocholate, 3-acetoxy-7,8-epoxylanostan-1-ol (Okoye et al. 2011)	Antibacterial activities of crude against *Bc*, *Ef*, *Ec*, *Sa*, *Kp*, *Mc*, *Pm*, *Ss* (Siwe Noundou et al. 2014); topical anti-inflammatory effects (Okoye et al. 2011)
*Annona muricata* Lin. (Annonaceae); 18681/SRF/Cam	Treatment of wounds and insomnia; antiparasitic, insecticidal (Rajeswari 2012)	Leaves (4.50 %), seeds (9.15 %), pericarp (5.17 %)	Epomuricenins-A and B, montecristin, cohibins-A and B, muridienins-1 and 2, muridienins-3 and 4, muricadienin and chatenaytrienins-1, 2 and 3 and sabadelin, murihexol, donhexocin, annonacin A and Annonacin B (Rajeswari 2012)	Antimicrobial activities of aqueous, ethanol and methanol extracts against *Sa*, *Vc*, *Ec*, *Se*, *Lv* and *On* (Vieira et al. 2010) and *Pv*, *Sp*, *Bs*, *St*, *Kp*, *Ea* (Rajeswari 2012), *Lb*, *Lp*, *Hv* (Rajeswari 2012), *Ec*, *Ea*, *Kp*, *Ps* (Dzotam et al. 2015)
*Canarium schweinfurthii* Engl. (Burceraceae); 16929/SRF/Cam	Treatment of malaria, constipation, diarrhea, rheumatism and sexually transmitted diseases (Koudou et al. 2005)	Fruits (0.78 %)	Saponins, cardiac glycosides, tannins, flavonoids and steroids (Ngbede et al. 2008)	Antimicrobial activities of EO against *Bc*, *Ef*, *Ec*, *Li*, *Se*, *Sd*, *Sa*, *Pm*, *Sc* and *Ca* (Obame et al. 2007)
*Colocasia esculenta* (L.) Schott (Araceae); 42352/HNC	Treatment scorpion and snake bite (Nakade et al. 2013), infectious diseases (Dzotam et al. 2015)	Leaves (6.25 %)	Quinones, alkaloids, saponins, tannins, phenols, terpenoids, glycosides and steroids (Nakade et al. 2013)	Antimicrobial activities of ethyl acetate extract against *St*, *Kp*, *Pa*, *Sp*, *Bs*, *Pv*, *Ec* (Nakade et al. 2013) aqueous and methanolic extracts: (Q) *Vspp* (Lee et al. 2010)
*Euphorbia prostata* W. Ait. (Euphorbiaceae) 33585/HNC	Treatment of bronchial ashma, diarrhea, skin diseases (Shrama and Tripathi 1983)	Whole plant (13.82 %)	Flavonoids, tannins and phenolic acid; gallic acid, apigenin, luteolin (Gupta 2011)	Crude extract has cardiac depresent and hypotensive actions (Shrama and Tripathi 1983), showed effects on early grades of hemorrhoids (Gupta 2011)
*Hibiscus esculentus*L. (Tiliaceae); 8537/SRF/Cam	Treatment of cancer, inflammation, ulcer, analgesic, hyperglycemia (Daly 1997; Uraku et al. 2010)	Fruits (2.98 %)	Alkaloids, polyphenols, flavonoids, triterpenes, sterols (Dzotam et al. 2015)	Antimicrobial activities of crude extract on *St*, *Shigella* and *Ec*, *Ea*, *Kp*, *Ps* (Dzotam et al. 2015; Nwaiwu et al. 2012)
*Moringa oleifera* Lam. (Moringaceae); 49178/HNC	Treatment of cancer, dental caries, syphilis, typhoid, diarrhea, epilepsy (Fuglie 1999), fever, HIV-AIDS (Abrams et al. 1993)	Leaves (3.95 %)	4-(4′-*O*-acetyl-α-l-rhamnopyranosyloxy)benzylisothiocyanate, 4-(-l-rhamnopyranosyloxy)benzylisothiocyanate, niazimicin, pterygospermin, benzylisothiocyanate and 4-(α-l-rhamnopyranosyloxy)benzylglucosinolate (Fahey 2005)	Antimicrobial activities of aqueous and ethanol extracts of seeds against *Sa*, *Vc*, *Ec*, *Se*, *Lv* and *On* (Viera et al. 2010)
*Pachypodanthium staudtii* Engl & Diels (Annonaceae), 23170 SFR/Cam	Treatment of cancer, Chest pain (Irvine 1961); bronchitis (Bouquet and Debray 1974) and oedema (Ngadjui et al. 1989).	Leaves (10 %), bark (9.4 %) and roots (6.25 %)	Pachypodol, 2,4,5-Trimethoxystyrene, Pachypophyllin, pachypostaudins A and B (Ngadjui et al. 1989); Sabinene, β-elemene, *E*-β-caryophyllene, β-selinene, β-bisabolene, δ-cadinene, 2,4,5-trimethoxy-1-vinylbenzene (Yapi et al. 2012).	Methanol extract against *Ec*, *Ea*, *Ecl*, *Kp*, *Ps* (Fankam et al. 2014)
*Passiflora edulis* Sims (Passifloraceae); 65104/HNC	Treatment of cancer, fungal infections, inflammation, insomnia and anxiety, antihypertensive (Ichimura et al. 2006), gastric trouble (Silva et al. 2006), antioxidant (Kannan et al. 2011)	Fruit (3.92 %); fruit pericarp (2.73 %)	Ionone-I, ionone-II, megastigma-5,8-dien-4-1, megastigma-5,8(*Z*)-diene-4-1, 4,4*a*-Epoxy-4, 4*a*-dihydroedulan, 3-hydroxyedulan, edulan-I, edulan-II, passifloric acid methyl ester (Kannan et al. 2011)	Antimicrobial activities of methanol extract against *Ec*, *Kp*, *Ea*, *Pa*, *Ps*, *Sa*, *Ef*, *Bs*, *Ec*, *Pv* and *St* (Kannan et al. 2011)
*Triumphetta pentandra* A.Rich. (Tiliaceae); 9014/SRF/Cam	Induce fertility and implantation of the fetus (Okoli et al. 2007; Ngondi et al. 2005), treat infectious diseases (Dzotam et al. 2015)	Leaves (5.50 %)	Triumfettamide, triumfettoside, heptadecanoic acid, β-sitosterol glucopyranoside, friedeline, lupeol, betuline, maslinic acid, 2-hydroxyoleanolicacid and the mixture of stigmasterol and β-sitosterol (Sandjo et al. 2008; Sandjo and Kuete 2013)	Antimicrobial activities of methanol extract against *Ec*, *Ea*, *Kp*, *Ps* (Dzotam et al. 2015)
*Xanthosoma mafaffa* (L.) Schott (Araceae); 18675/SRF/Cam	Treatment of infectious diseases; osteoporosis (Dzotam et al. 2016; Cancer in Africa 2012)	Leaves (4.30 %)	Polyphenols, coumarins, tannins, triterpenes, sterols, saponins (Dzotam et al. 2016)	Antimicrobial activities of methanol extract against *Ec*, *Ea*, *Kp* (Dzotam et al. 2016)

EO: essential oil; *Bc*: *Bacillus cereus*; *Bs*: *Bacillus subtilis*; *Ca*: *Candida albicans*; *Ec*: *Escherichia coli*; *Ea*: *Enterobacter aerogenes*; *Ecl*: *Enterobacter cloacae*; *Ef*: *Enterococcus faecalis*; HIV-AIDS: human immunodeficiency virus-acquired immuno deficiency syndrome; *Hv*: *Herpes virus*; *Kp*: *Klebsiella pneumoniae*; *Lb*: *Leishmania braziliensis*; *Lp*: *Lieshmaniapanamensis*; *Lv*: *Litopenaeusvannmaei*; *Mc*: *Moraxella catarrhalis*; *On*: *Oreochromis nicoticus*; *Pa*: *Pseudomonas aeruginosa*; *Li*: *Listeria innocua*; *Pm*: *Proteus mirabilis*; *Pv*: *Proteus vulgaris*; *Ps*: *Providencia stuartii*; *Sa*: *Staphylococcus aureus*; *Sc*: *Staphylococcus camorum*; *Sd*: *Shigelladysenteriae*; *Se*: *Salmonella enterica*; *Ss*: *Staphylococcus saprophyticus*; *Sp*: *Streptococcus pyogenes*; *St*: *Salmonella typhi*; *Vc*: *Vibrio cholerae*; *Vspp*: Vibrio species; underline: disease states bearing relevance to cancer or cancer-like symptoms

^a^(HNC): Cameroon National Herbarium; (SRF/Cam): Société des Réserves Forestières du Cameroun

^b^Yield calculated as the ratio of the mass of the obtained methanol extract/mass of the plant powder

### Chemicals

Doxorubicin 98.0 % and vinblastine ≥96 % from Sigma-Aldrich (Munich, Germany) were provided by the University Pharmacy of the Johannes Gutenberg University (Mainz, Germany), dissolved in phosphate buffer saline (PBS; Invitrogen, Eggenstein, Germany) at a concentration of 10 mM and used as positive control drugs. Geneticin >98 % (Sigma-Aldrich), stored at a stock concentration of 72.18 mM was used to maintain the resistance patterns of MDR carcinoma cell lines.

### Cell cultures

The cell lines used in the present study included drug-sensitive leukemia CCRF–CEM and multidrug-resistant P-glycoprotein-over-expressing subline CEM/ADR5000 cells (Efferth et al. 2003a; Kimmig et al. 1990; Gillet et al. 2004), breast cancer MDA-MB-231-pcDNA3 cells and its resistant subline MDA-MB-231-*BCRP* clone 23 (Doyle et al. 1998), colon cancer HCT116 (*p53*^+*/*+^) cells and its knockout clone HCT116 (*p53*^−*/*−^), glioblastoma U87MG cells and its resistant subline U87MG.Δ*EGFR* (Kuete et al. 2013a, b; Dzoyem et al. 2013). Leukemia CCRF–CEM and CEM/ADR5000 cells were maintained in RPMI 1640 medium (Invitrogen) supplemented with 10 % fetal calf serum in a humidified 5 % CO_2_ atmosphere at 37 °C.

Sensitive and resistant cells were kindly provided by Dr. J. Beck (Department of Pediatrics, University of Greifswald, Greifswald, Germany). Breast cancer cells transduced with control vector (MDA-MB-231-pcDNA3) or with cDNA for the breast cancer resistance protein *BCRP* (MDA-MB-231-*BCRP* clone 23) were maintained under standard conditions as described above for CCRF–CEM and CEM/ADR5000 cells. Human wild-type HCT116 (*p53*^+*/*+^) colon cancer cells as well as knockout clones HCT116 (*p53*^−^*/*^−^) derived by homologous recombination were a generous gift from Dr. B. Vogelstein and H. Hermeking (Howard Hughes Medical Institute, Baltimore, MD). Human glioblastoma multiforme U87MG cells (non-transduced) and U87MG cell line transduced with an expression vector harboring an epidermal growth factor receptor (*EGFR*) gene with a genomic deletion of exons 2 through 7 (U87MG.Δ*EGFR*) were kindly provided by Dr. W. K. Cavenee (Ludwig Institute for Cancer Research, San Diego, CA). MDA-MB-231-*BCRP*, U87MG.Δ*EGFR* and HCT116 (*p53*^−^*/*^−^) were maintained in DMEM medium containing 10 % FBS (Invitrogen) and 1 % penicillin (100 U/mL)-streptomycin (100 μg/mL) (Invitrogen) and were continuously treated with 800 ng/mL and 400 µg/mL geneticin, respectively. The multidrug resistance profile of these cell lines has been reported (Doyle et al. 1998). Human liver hepatocellular carcinoma HepG2 and the AML 12 normal heptocytes were obtained from ATCC (USA). The above medium without geneticin was used to maintained MDA-MB-231, U87MG, HCT116 (*p53*^+*/*+^), HepG2 and AML 12 cell lines. The cells were passaged twice weekly. All experiments were performed with cells in the logarithmic growth phase.

### Resazurin reduction assay

The cytotoxicity of the tested samples was performed by resazurin reduction assay as previously described (Kuete et al. 2013b; O’Brien et al. 2000). The assay is based on reduction of the indicator dye, resazurin, to the highly fluorescent resorufin by viable cells. Non-viable cells rapidly lose the metabolic capacity to reduce resazurin and thus produced no fluorescent signal. Briefly, adherent cells were detached by treatment with 0.25 % trypsin/EDTA (Invitrogen) and an aliquot of 1 × 10^4^ cells was placed in each well of a 96-well cell culture plate (Thermo Scientific, Germany) in a total volume of 200 µL. Cells were allowed to attach overnight and then were treated with different concentrations of the studied sample. For suspension cells, aliquots of 2 × 10^4^ cells per well were seeded in 96-well-plates in a total volume of 100 µL. The studied sample was immediately added in varying concentrations in an additional 100 µL of culture medium to obtain a total volume of 200 µL/well. After 24 or 48 h, 20 µL resazurin (Sigma-Aldrich, Germany) 0.01 % w/v in ddH_2_O was added to each well and the plates were incubated at 37 °C for 4 h. Fluorescence was measured on an Infinite M2000 Pro™ plate reader (Tecan, Germany) using an excitation wavelength of 544 nm and an emission wavelength of 590 nm. Each assay was done twice, with six replicates each. The viability was evaluated based on a comparison with untreated cells. IC_50_ values representing the sample’s concentrations required to inhibit 50 % of cell proliferation were calculated from a calibration curve by linear regression using Microsoft Excel (Kuete et al. 2011a; Dzoyem et al. 2012). In a preliminary step, all samples were tested against the sensitive CCRF–CEM cells at various concentrations ranging from 0.16 to 80 µg/mL (crude extracts) or 0.08 to 10 µg/mL (doxorubicin), and samples displaying IC_50_ values below 20 µg/mL were further investigated in 8 other tumor cell lines as well as in normal AML12 hepatocytes. Doxorubicin was used as positive control, while dimethylsulfoxide (DMSO) used to dissolve the samples was used as negative control. The highest concentration of DMSO was less than 0.4 %.

### Flow cytometry for cell cycle analysis and detection of apoptotic cells

Extracts from *Passiflora edulis* fruit (PEF), *Annona muricata* leaves (AML), *Annona muricata* seeds (AMS) that displayed the best cytotoxicity as well as doxorubicin were used to treat CCRF–CEM cells (1 × 10^6^) at their IC_50_ values. Thus, CCRF–CEM cells were cultured in RPMI medium as described above, in the presence of each sample at a concentration corresponding to the IC_50_ values obtained in the cell line. The cell cycle was then analyzed after incubation for 24, 48 and 72 h. All reagents, experimental conditions and apparatus were identical to those previously reported (Kuete et al. 2013a; Dzoyem et al. 2013). Briefly, cell cycle analysis was performed by flow cytometry using Vybrant^®^ DyeCycle™ (Invitrogen, Darmstadt, Germany). Cells were measured after Vybrant^®^ DyeCycle™ Violet staining (30 min at 37 °C) on a LSR-Fortessa FACS analyzer (Becton–Dickinson, Heidelberg, Germany) using the violet laser. Vybrant^®^ DyeCycle™ Violet stain was measured with 440 nm excitation. Cytographs were analyzed using FlowJo software (Celeza, Switzerland). All experiments were performed at least in triplicate.

### Caspase-Glo 3/7, caspase-Glo 8 and caspase-Glo 9 assay

The influence of extracts on caspase 3/7, caspase 8 and caspase 9 activity in leukemia CCRF–CEM cell line was detected using Caspase-Glo 3/7, Caspase-Glo 8 and Caspase-Glo 9 Assay kits (Promega, Germany). Cells cultured in RPMI medium were seeded in 96-well plates and treated with the sample (2 × IC_50_; IC_50_) or DMSO (solvent control). After 6 h incubation in a humidified 5 % CO_2_ atmosphere at 37 °C, 100 µL of caspase reagent were added to each well, mixed and incubated for 1 h at room temperature. Luminescence was measured using well Infinite M2000 Pro™ instrument (Tecan). Caspase activity was expressed as percentage relative to the untreated control (Kuete et al. 2014b).

### Analysis of mitochondrial membrane potential (MMP)

The effects of extracts on the MMP were analyzed by 5,5′,6,6′-tetrachloro-1,1′,3,3′-tetraethylbenzimidazolylcarbocyanine iodide) (JC-1; Biomol, Germany) staining (Kuete et al. 2013c). JC-1 is a dye that can selectively enter into mitochondria and exhibits an intense red fluorescence in healthy mitochondria with normal membrane potentials. In cells with reduced MMP, the red fluorescence disappears. Briefly, 1 × 10^6^ CCRF–CEM cells treated at different concentrations with PEF, AML, AMS or vinblastine for 24 h were incubated with JC-1 staining solution according to the manufacturer`s protocol for 30 min. Subsequently, cells were measured in a LSR-Fortessa FACS analyzer (Becton–Dickinson). For each sample, 1 × 10^4^ cells were counted. The JC-1 signal was measured with 561 nm excitation (150 mW) and detected using a 586/15 nm bandpass filter. The samples signal was analyzed with 640 nm excitation (40 mW) and detected using a 730/45 nm bandpass filter. All parameters were plotted on a logarithmic scale. Cytographs were analyzed using FlowJo software (Celeza, Switzerland). All experiments were performed in triplicate.

### Measurement of reactive oxygen species (ROS) by flow cytometry

The 2′,7′-Dichlorodihydrofluorescein diacetate (H_2_DCFH-DA) (Sigma-Aldrich, Germany) is a probe used for the highly sensitive and quantifiable detection of ROS. The non-fluorescent H_2_DCFH-DA diffuses into the cells and is cleaved by cytoplasmic esterases into 2′,7′-dichlorodihydrofluorescein (H_2_DCF) which is unable to diffuse back out of the cells. In the presence of hydrogen peroxide, H_2_DCF is oxidized to the fluorescent molecule dichlorofluorescein (DCF) by peroxidases. The fluorescent signal emanating from DCF can be measured and quantified by flow cytometry, thus providing an indication of intracellular ROS concentration (Kuete et al. 2011c; Bass et al. 1983; Cossarizza et al. 2009). Briefly, 2 × 10^6^ CCRF–CEM cells were resuspended in PBS and incubated with 2 µM H_2_DCFH-DA for 20 min in the dark. Subsequently, cells were washed with PBS and resuspended in RPMI 1640 culture medium containing different concentrations of PEF, AML, AMS or DMSO (solvent control), or hydrogen peroxide (H_2_O_2_; positive control). After 24 h of incubation, cells were washed and suspended in PBS. Subsequently cells were measured in a FACSCalibur flow cytometer (Becton–Dickinson, Germany). For each sample 1 × 10^4^ cells were counted. DCF was measured at 488 nm excitation (25mW) and detected using a 530/30 nm bandpass filter. All parameters were plotted on a logarithmic scale. Cytographs were analyzed using FlowJo software (Celeza, Switzerland). All experiments were performed in triplicate.

## Results

### Cytotoxicity of the studied samples

In this study, we first screened the cytotoxicity of 17 crude extracts belonging to 11 plants towards drug-sensitive CCRF–CEM leukemia cells. The results are shown in Table 2. All tested extracts had IC_50_ values below 80 µg/mL. Ten extracts from five plants including *Alchornea floribunda* bark (AFB), *Annona muricata* fruit pericarp (AMP), leaves (AML) and seeds (AMS), *Euphorbia prostata* whole plant (EPW), *Pachypodanthium staudtii* bark (PSB), leaves (PSL) and roots (PSR), and *Passiflora edulis* fruit pericarp (PEP) and fruit (PEF) displayed IC_50_ values below 20 µg/mL in CCRF–CEM cells (Table 2). These extracts were further selected for IC_50_ determination towards a panel of sensitive and MDR cell lines. The results summarized in Table 3 indicate that all selected extracts were also active against P-glycoprotein-over-expressing CEM/ADR5000 leukemia cells with IC_50_ values below 40 µg/mL. IC_50_ values ranged from 10.13 µg/mL (towards CEM/ADR5000 cells) to 72.01 µg/mL (on resistant colon carcinoma HCT116 (*p53*^−*/*−^) cells) for PSR, from 14.97 µg/mL (on CEM/ADR5000 cells) to 65.68 µg/mL (against HCT116 (*p53*^−*/*−^) cells) for PSB, from 18.21 µg/mL (against CEM/ADR5000 cells) to 65.21 µg/mL (on HCT116 (*p5*^+*/*+^) cells) for PSL and from 0.11 µg/mL (towards CCRF–CEM cells) to 108 µg/mL (against CEM/ADR5000 cells) for doxorubicin in the 8 other cancer cell lines studied. Apart from extract from *P. staudtii*, other extracts were less active on carcinoma cells including normal AML12 hepatocytes, with IC_50_ values above 80 µg/mL. Collateral sensitivity (or hypersensitivity: higher toxicity to resistant than to sensitive cells with a degree of resistance below 1) (Kuete et al. 2013a) was observed in CEM/ADR5000 cells to PSB (degree of resistance of 0.87-fold) and PSR (0.59-fold) (Table 3). Hypersensitivity of resistant carcinoma cells was also recorded in many cases to PSL, PSB or PSR even though they were moderately active. However, if cross-resistance of CEM/ADR5000 cells to the tested extracts were observed, the degrees of resistance were in all cases lower than that of doxorubicin (Table 3). AMS, AML and PEF had IC_50_ values below 1 and 10 µg/mL in sensitive CCRF/CEM cells and it resistant subline CEM/ADR5000 cells respectively; they were subsequently selected for mechanistic studies.

**Table 2:** IC_50_ values of the tested plant extracts towards leukemia CCRF–CEM cells and as determined by the resazurin assay

Tested plant and parts		IC_50_ values (µg/mL)
Plants	Parts	
*Alchornea floribunda*	Bark (AFB)	*18.88* ± *1.65*
	Leaves	46.00 ± 4.26
*Annona muricata*	Fruit pericarp (AMP)	*4.58* ± *0.25*
	Leaves (AML)	*0.57* ± *0.02*
	Seeds (AMS)	*0.36* ± *0.03*
*Canarium schweinfurthii*	Fruit	38.62 ± 3.69
*Colocasia esculenta*	Leaves	38.19 ± 4.39
*Euphorbia prostata*	Whole plant (EPW)	*18.59* ± *1.12*
*Hibiscus esculentus*	Fruit	60.79 ± 7.04
*Moringa oleifera*	Leaves	29.79 ± 1.26
*Pachypodanthium staudtii*	Bark (PSB)	*17.22* ± *1.16*
	Leaves (PSL)	*13.59* ± *1.12*
	Roots (PSR)	*17.62* ± *1.18*
*Passiflora edulis*	Fruit pericarp (PEP)	*3.41* ± *0.55*
	Fruit (PEF)	*0.69* ± *0.13*
*Triumphetta pentandra*	Leaves	36.28 ± 2.84
*Xanthosoma mafaffa*	Leaves	43.20 ± 0.99
Doxorubicin		*0.11* ± *0.03*

In italics: significant cytotoxic effect

**Table 3:** Cytotoxicity of the tested extracts and doxorubicin towards sensitive and drug-resistant cancer cell lines and normal cells as determined by the resazurin assay

Cell lines	Samples, IC_50_ values in µg/mL and degrees of resistance^a^ (in bracket)										Doxorubicin
	AFB	AML	AMS	AMP	EPW	PSL	PSB	PSR	PEF	PEP	
CEM/ADR5000	29.49 ± 1.77 (1.56)	*5.25* ± *0.38* (9.29)	*6.65* ± *0.22* (18.47)	23.70 ± 1.64 (5.17)	37.00 ± 2.17 (1.99)	*18.21* ± *1.45* (1.34)	*14.97* ± *0.97* (0.87)	*10.13* ± *0.88* (0.59)	*8.20* ± *1.02* (11.88)	*18.40* ± *1.42* (5.40)	108.00 ± 7.92 (975.60)
MDA-MB-231-*pcDNA*	>80	>80	>80	>80	>80	52.08 ± 4.98	52.66 ± 6.03	37.19 ± 2.74	>80	>80	*0.61* ± *0.15*
MDA-MB-231-*BCRP* Degree of resistance	>80	>80	>80	>80	>80	61.98 ± 4.31 (1.19)	47.27 ± 3.76 (0.90)	46.92 ± 4.89 (1.26)	>80	>80	*4.33* ± *0.26* (7.12)
HCT116 (*p53* ^+*/*+^)	>80	>80	>80	>80	>80	65.21 ± 7.15	34.35 ± 1.99	28.66 ± 1.62	>80	>80	*0.78* ± *0.16*
HCT116 (*p53* ^−*/*−^) Degree of resistance	>80	>80	>80	>80	>80	56.97 ± 4.09 (0.87)	65.68 ± 4.80 (1.91)	72.01 ± 5.26 (2.51)	>80	>80	*2.25* ± *0.04* (2.88)
U87MG	>80	>80	>80	>80	>80	65.21 ± 5.79	52.46 ± 5.22	24.80 ± 1.36	>80	>80	*0.59* ± *0.08*
U87MG.Δ*EGFR* Degree of resistance	>80	>80	>80	>80	>80	68.65 ± 3.48 (1.05)	58.70 ± 3.67 (1.12)	46.91 ± 3.01 (1.89)	>80	>80	*3.38* ± *0.32* (5.76)
HepG2	38.69 ± 3.08 (>802.07)	>80	>80	>80	>80	46.98 ± 3.17 (>1.70)	36.39 ± 3.08 (0.62)	37.56 ± 2.17 (0.80)	>80	>80	*2.12* ± *0.52* (>37.74)
AML12 Degree of resistance	>80	>80	>80	>80	>80	>80	>80	>80	>80	>80	>80

^a^The degree of resistance was determined as the ratio of IC_50_ value in the resistant divided by the IC_50_ in the sensitive cell line; CEM/ADR5000, MDA-MB-231-*BCRP*, HCT116 (*p53* ^−*/*−^), U87MG.Δ*EGFR* and AML12 were used as the corresponding resistant counterpart for CCRF–CEM (Table 1), MDA-MB-231-*pcDNA*, HCT116 (*p53* ^+*/*+^), U87MG and HepG2 respectively; the tested methanol extracts were from AFB: *Alchornea floribunda* bark; AML: *Annona muricata l*eaves; AMS: *Annona muricata* seeds; AMP: *Annona muricata* fruit pericarp; EPW: *Euphorbia prostata* whole plant; PSL: *Pachypodanthium staudtii* leaves; PSB: *Pachypodanthium staudtii* bark; PSR: *Pachypodanthium staudtii* roots; PEF: *Passiflora edulis* fruit; PEP: *Passiflora edulis* fruit pericarp; in italics: significant activity

### Cell cycle distribution and apoptosis

**Fig. 1:**
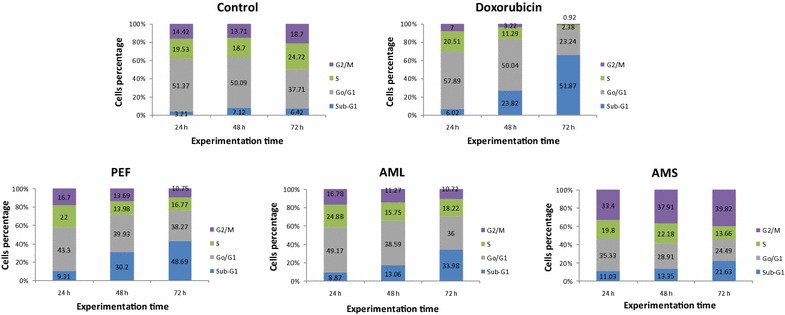
Cell cycle distribution of CCRF–CEM leukemia cells treated with extracts from PEF, AML and AMS or doxorubicin. PEF, AML and AMS were tested at 0.69, 0.57 and 0.36 and 8.02 µg/mL respectively while doxorubicin was tested at 0.11 µg/mL corresponding to their IC_50_

The best extracts (AMS, AML and PEF) as well as doxorubicin were used to treat CCRF–CEM cells at their IC_50_ values, and the cycle distribution was analyzed. Results depicted in Fig. 1 show dose-dependent and significant modifications of the cell cycle phases after treatment of cells with all samples. Both PEF and AML induced cell cycle arrest in G0/G1 phase while AMS induced cell cycle arrest in S-phase. After treatment with these three extracts, CCRF–CEM cells underwent apoptosis with dose-dependent increases in sub-G0/G1 phase. The percentages of cells in sub-G0/G1 phase varied from 9.31 % (in 24 h) to 48.69 % (72 h), from 8.87 % (in 24 h) to 33.98 % (72 h) and from 11.03 % (24 h) to 21.63 % (72 h) after PEP, AML and AMS treatments respectively, while doxorubicin increased apoptosis in a range of 6.02 % (24 h) to 51.87 % (72 h). The highest percentage of sub-G0/G1 phase in non-treated cells was only 6.42 % after 72 h.

### Effects on the activity of caspases, MMP and ROS

**Fig. 2:**
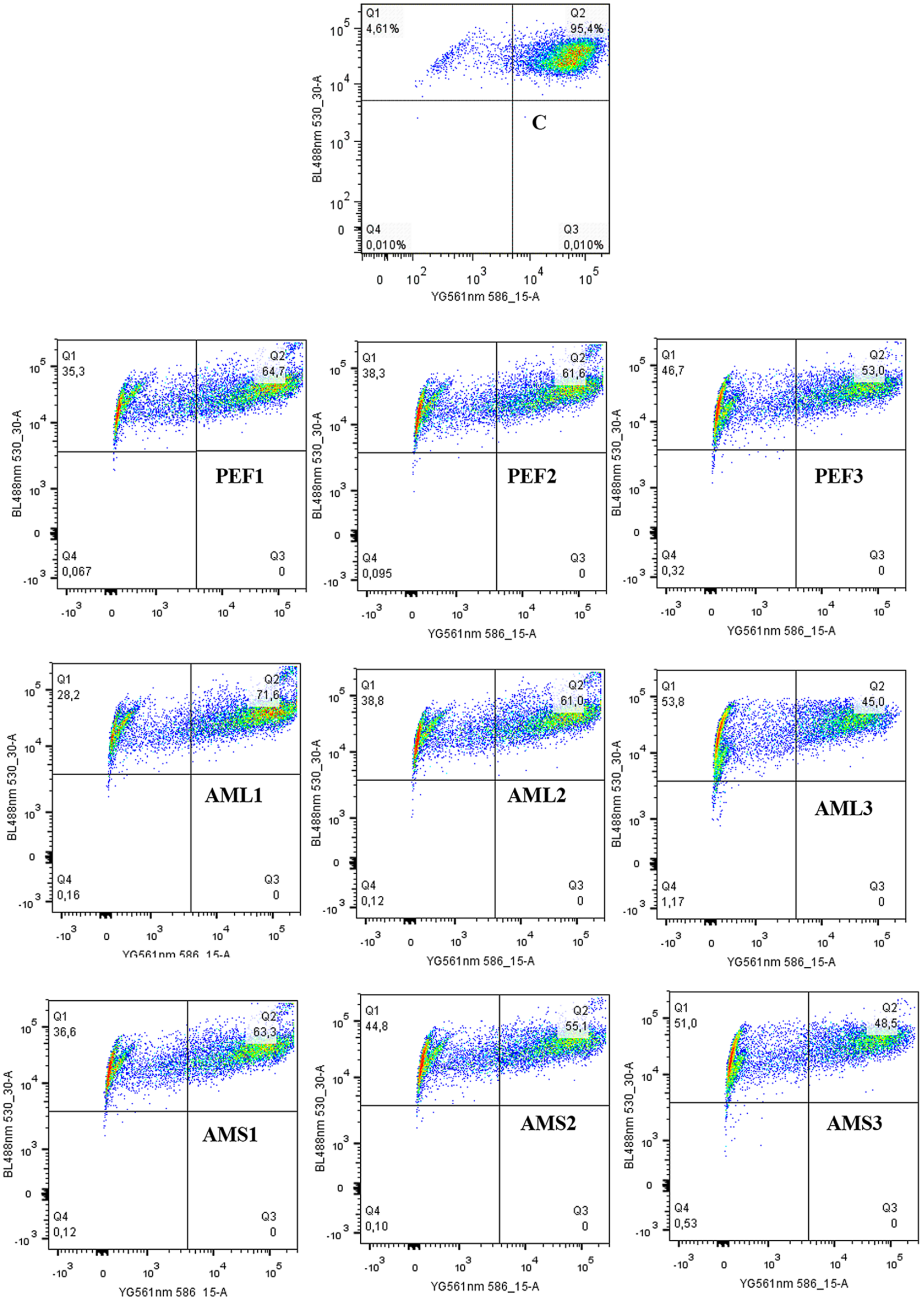
Effect of PEF, AML and AMS on the mitochondrial membrane potential in CCRF–CEM cells. *C* control; PEF was tested at 24 h at 0.35 µg/mL (PEF1), 0.69 µg/mL (PEF2), and 1.38 µg/L (PEF3) while AML was tested at 0.29 µg/mL (AML1), 0.57 µg/mL (AML2), and 1.14 µg/mL (AML3) and AMS was tested at 0.18 µg/mL (AML1), 0.36 µg/mL (AML2), and 0.72 µg/mL (AML3) corresponding to 1/2-fold, IC_50_ and 2-fold IC_50_. Data for the positive control, vinblastine in similar experimental conditions were previously reported (Kuete et al. 2013b); Loss of MMP (Q1), intact cells (Q2), ruptured cell membrane (Q3 and Q4)

After treating CCRF–CEM cells for 6 h at different concentrations of PEF, AML and AMS, no changes of caspase 3/7, caspase 8 and caspase 9 activities were observed. No increase in ROS production was also not found in CCRF–CEM cells treated with the three extracts (data not shown). PEF, AML and AMS induced significant MMP loss in the respective ranges of 35.3 % (1/2-fold IC_50_ treatment) to 46.7 % (2-fold IC_50_), 28.2 % (1/2-fold IC_50_) to 53.8 % (2-fold IC_50_) and 36.6 % (1/2-fold IC_50_) to 51.0 % (2-fold IC_50_) (Fig. 2). A 48.6 % loss of MMP at 2-fold IC_50_ of vinblastine was previously reported under similar experimental conditions in CCRF–CEM cells (Kuete et al. 2013a).

## Discussion

According to the U.S. National Cancer Institute (NCI) plant screening program, plant extracts with IC_50_ values below of 20 µg/mL following incubation between 48 and 72 h (Boik 2001) are recognized as potential cytotoxic substances. In the present study, multi-factorial drug-resistant cancer cell lines such as leukemia CEM/ADR5000 cells over-expressing P-gp, breast adenocarcinoma MDA-MB-231-*BCRP* clone 23 expressing BCRP, EGFR-transfected U87MG.Δ*EGFR* glioblastoma cells and p53 knockout HCT116 (*p53*^−*/*−^) colon cancer cells (Efferth et al. 2003a; Kuete et al. 2013a, b, 2014c; Kimmig et al. 1990; Gillet et al. 2004; Doyle et al. 1998) were used to determine the cytotoxicity the selected plant extracts. In the first step of the investigations, we carried out a preliminary assays with the sensitive leukemia CCRF–CEM cells. In regards to the NCI threshold, AFB, AMP, AML, AMS, EPW, PSB, PSL, PSR, PEP and PEF (Table 2) displaying IC_50_ values below 20 µg/mL were selected and further tested on a panel of 8 other cell lines. Interestingly, the P-gp over-expressing leukemia CEM/ADR5000 was also sensitive to most of the extracts with IC_50_ value below 20 µg/mL obtained with AML, AMS, PSL, PSB, PSR, PEF and PEP. This suggests that these extracts can be used to manage hematological cancers including resistant phenotypes. Data obtained with AML, AMS and PEP are very interesting as they displayed IC_50_ values below 10 µg/mL in the resistant CEM/ADR5000 cells and even below 1 µg/mL in its sensitive counterpart CCRF–CEM cells. Nonetheless, they were not active in carcinoma cells, clearly indicating their selectivity to leukemia cells. Alteration of MMP has been reported as a mode of apoptosis induction of plant extracts (Kuete and Efferth 2015). AML, AMS and PEP induced MMP loss but no caspase activation nor increase ROS production. Hence, MMP is the main mode of induction of apoptosis of AML, AMS and PEP in CCRF–CEM cells as observed in this study.

To the best of our knowledge, the cytotoxicty of the five most active plants, *Alchornea floribunda*, *Annona muricata*, *Euphorbia prostata*, *Pachypodanthium staudtii* and *Passiflora edulis* towards the cell line panel tested in this study is being reported for the first time. Nevertheless, the leaves ethanol extract of *Annona muricata* was reported to have antiproliferative effect against leukemia HL-60 cells with an IC_50_ value of 14 µg/mL, and also induced apoptosis through the loss of MMP with G0/G1 phase cell arrest (Pieme et al. 2014). This is in accordance with data reported herein. The ethyl acetate extract of the leaves of this plant harvested in Malaysia was also found active against colon carcinoma HCT-116 and HT-29 cells with the respective IC_50_ values of 11.43 and 8.98 µg/mL (Zorofchian Moghadamtousi et al. 2014). In the present study, IC_50_ were not detected at up to 80 µg/mL, either indicating that the active constituents of the plant against carcinoma cells might not be well extracted with methanol or that the geographic distribution influences the cytotoxic potential of the plant. Also the methanol extracts of the leaves and fruits of *Passiflora edulis* harvested in Egypt were screened at 100 µg/mL against HCT-116 cells, HepG2 cells as well as against the breast carcinoma MCF-7 cells and lung carcinoma A-549 cells; As results, less than 50 % growth inhibition was recorded (Moustafa et al. 2014), coroborating the low activity obtained with various parts of this plant against carcinoma cells.

## Conclusions

In this study, ten extracts from five medicinal plants, *Alchornea floribunda*, *Annona muricata*, *Euphorbia prostata*, *Pachypodanthium staudtii* and *Passiflora edulis* had good cytotoxicity against CCRF–CEM leukemia cells and its resistant subline CEM/ADR5000 cells. Their selectivity to these two cell lines, indicates that they can be sources for the development of novel anticancer drugs to fight leukemia. AML, AML and PEF were the most cytotoxic extracts and induced apoptosis in CCRF–CEM cells mediated by loss of MMP. Further phytochemical investigations of these extracts will be done to isolate their active constituents.

## Abbreviations

ABC: adenosine triphosphate-binding cassette
AFB: *Alchornea floribunda* bark
AML: *Annona muricata l*eaves
AMP: *Annona muricata* fruit pericarp
AMS: *Annona muricata* seeds
BCRP: breast cancer resistance protein
DCF: dichlorofluorescein
DMSO: dimethylsufoxide
EGFR: epidermal growth factor receptor
EPW: *Euphorbia prostata* whole plant
H_2_DCFH-DA: 2′,7′-dichlorodihydrofluorescein diacetate
IC_50_: inhibitory concentration 50 %
JC-1: 5,5′,6,6′-tetrachloro-1,1′,3,3′-tetraethylbenzimidazolylcarbocyanine iodide
MDR: multi-drug resistant
MMP: mitochondrial membrane potential
PBS: phosphate buffer saline
PEF: *Passiflora edulis* fruit
PEP: *Passiflora edulis* fruit pericarp
P-gp: P-glycoprotein
PSB: *Pachypodanthium staudtii* bark
PSL: *Pachypodanthium staudtii* leaves
PSR: *Pachypodanthium staudtii* roots
ROS: reactive oxygen species
